# EFFECT OF AN INTERPROFESSIONAL STUDENT-LED THERAPEUTIC ACTIVITY PROGRAM ON OUTCOMES OF DEMENTIA PARTICIPANTS

**DOI:** 10.1093/geroni/igae098.2355

**Published:** 2024-12-31

**Authors:** Kerry Jordan, Melissa Allen, Michael Gallagher, January Schultz, Richelle Weese, Suzette Marks

**Affiliations:** University of Central Arkansas, Conway, Arkansas, United States; University of Central Arkansas, Conway, Arkansas, United States; University of Central Arkansas, Conway, Arkansas, United States; University of Central Arkansas, Conway, Arkansas, United States; University of Central Arkansas, Conway, Arkansas, United States; University of Central Arkansas, Conway, Arkansas, United States

## Abstract

Despite demonstrated effectiveness, access to nonpharmacological interventions to help maintain cognition and function in people living with Alzheimer’s Disease and Related Dementias (ADRD) is severely limited. The Inter-professional Student led Therapeutic Activity program (STAP) improves access by engaging health science students to perform such activities as part of their clinical. STAP involves weekly, 3.5-hour sessions over 12 weeks per semester. During sessions, students from a variety of disciplines engage participants in evidence-based activities including group exercise, physical therapy, cognitive therapy, cognitive stimulation, and socialization. We aimed to evaluate initial effect of STAP on cognitive status, quality of life, and function of people with mild to moderate ADRD. Initial pre and post test data obtained over two 12- week intervention sessions demonstrate no statistically significant change in cognition using the Arizona Battery of Cognitive Communication Disorders Scale (ABCD-2) N12 (t -.707, p =.480); Quality of life using the Dem-QOL N11 (t.831, p =.406); and function using the 30-Second Chair Rise N18 (t-.087, p =.932), Timed up and Go Test (TUG) (t.962, p =.35), and 10 Meter Walk Test (t -1.932, p =.07). There was a significant improvement between pre and post test scores in independent activities of daily living using the Texas function Living Scale (TFLS) N11(t 2.0, p = 0.046). Results indicate that, in the short term, STAP may be beneficial in maintaining cognition, function and quality of life in people with mild to moderate ADRD. Further study of long term results is recommended.

